# Liver Transplantation during Pregnancy for Acute Liver Failure due to HBV Infection: A Case Report

**DOI:** 10.1155/2013/356560

**Published:** 2013-12-09

**Authors:** Nina Kimmich, Philipp Dutkowski, Franziska Krähenmann, Beat Müllhaupt, Roland Zimmermann, Nicole Ochsenbein-Kölble

**Affiliations:** ^1^Department of Obstetrics, University Hospital of Zurich, Frauenklinikstrasse 10, 8091 Zurich, Switzerland; ^2^Department of Surgery and Transplantation, University Hospital of Zurich, Frauenklinikstrasse 10, 8091 Zurich, Switzerland; ^3^Department of Gastroenterology and Hepatology, University Hospital of Zurich and Swiss HPB Center, Switzerland

## Abstract

Acute hepatic failure during pregnancy is a life-threatening situation for the mother and fetus and might need a super-urgent liver transplantation. Many pregnancies with positive outcomes are reported after a previous liver transplantation before the pregnancy, but only a few of them are mentioned with transplantation during pregnancy. In these few cases, fetal outcome is mostly adverse. Experience with liver failure during pregnancy and its management is still deficient and needs to be approved. For sure, patients need to be treated in highly qualified centers in a multidisciplinary approach. We present a case of successful super-urgent liver transplantation during the second trimester of pregnancy after acute hepatic failure due to an acute hepatitis B infection with positive maternal and fetal outcome. Liver transplantation during pregnancy due to an acute liver failure can be a life-saving procedure for the mother and fetus. An early initiated maternal therapy with antiviral drugs and immunoglobulins seems to be safe and able to prevent fetal infection and immunosuppressive therapy after transplantation seems to be well tolerated. Nevertheless, fetal outcome differs widely and long-term outcome is deficiently known.

## 1. Introduction

Acute hepatic failure is always a life-threatening situation. Sometimes, the only solution solving the problem is a super-urgent liver transplantation. An ongoing pregnancy aggravates such a situation, as two people's lives are involved, the one of the mother and the fetus. Many pregnancies with positive outcomes are reported after liver transplantation before getting pregnant, but only a few cases are mentioned with transplantation during pregnancy [1–21].

## 2. Case Presentation

**Figure 1:**
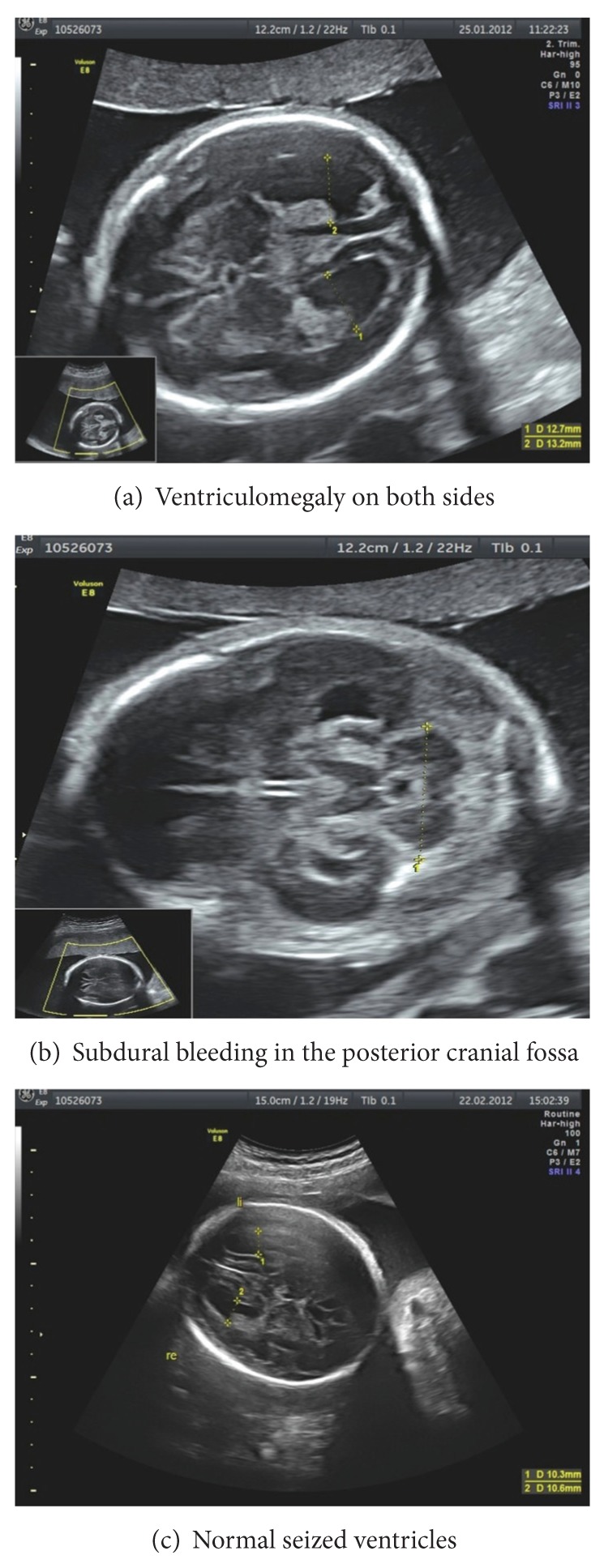
Sonographic images.

A 30-year-old primigravida at 22 0/7 gestational weeks (GW) was admitted to our hospital in suspicion of an acute hepatitis B infection. She had presented herself in another hospital with slight nausea and vomiting, pruritus, slight jaundice, dark urine, a fairly affected overall condition, and a positive blood test for acute hepatitis B infection. The source of the infection remained unknown. Her blood pressure, heart rate, and oxygen saturation were normal, as well as her urine analysis besides an elevated bilirubin level. She showed no signs of edema or hyperreflexia but slight jaundice and icteric sclera. Her cardiac and pulmonary status was unaffected, and her temperature was elevated to 38.8°C. Her laboratory work-up showed a progressive hepatic failure (Table 1). HELLP syndrome, intoxication with acetaminophen, Wilson's disease, hepatic neoplasia, acute fatty liver of pregnancy, autoimmune hepatitis, and alpha-1-antitrypsin deficiency as other potential etiologies of acute liver failure were excluded by laboratory testing. The infection screening revealed an acute hepatitis B infection with a viral load of 170.000.000 IU/mL. Other infections as hepatitis A, hepatitis C, HIV, cytomegalovirus (CMV), epstein barr virus, herpes simplex virus, varicella zoster virus, syphilis, toxoplasmosis, and parvovirus B19 could be excluded. Her personal medical history showed only an infection with chlamydia trachomatis in early pregnancy, which had been treated with azithromycin. Her family history revealed no cases of known hepatitis B infections. The fetus was sonographically unaffected and well developed according to her gestational age at admission. A multidisciplinary team of hepatologists, surgeons, and obstetricians took care of the patient. An antiviral therapy with tenofovir, 245 mg orally once a day, was initiated immediately. But the patient, who dearly wanted to keep the pregnancy, had to be added to the super-urgent liver transplantation list two days after admission according to Clichy criteria because of rapid progression of hepatic failure and encephalopathy (lab MELD 33) (Table 1). After graft allocation and within 24 hours, an orthotopic liver transplantation with cava preserving technique (piggy back) and intermittent portocaval shunt was performed to avoid any cava clamping during transplantation. The operation was uneventful (six-hour surgery, transfusion of 2 U red blood cells, and low pressors). Intraoperatively, an additional treatment with hepatitis B immunoglobulin was started and continued for 10 days in a dose of 10.000 IU per day intravenously. Thereafter, it was continued to maintain the anti-HBs titer >100 IU/mL. Postoperatively, treatment with tenofovir was continued until 28 6/7 GW. Afterwards, it was changed to lamivudine 100 mg p.o. daily because of an increase of liver enzymes. Immunosuppression consisted of corticosteroids and tacrolimus. The corticosteroids were applied intravenously for the first five days postoperatively in declining doses of methylprednisolone from 250 to 40 mg, followed by decreasing doses of prednisolone orally from 20 mg to 5 mg until delivery. Tacrolimus was applied orally in doses between 5 mg and 12 mg with the goal to achieve a blood level of six to eight ng/mL. On the first postoperative day, the patient could be extubated without any problems and recovered quickly. The histopathological examination of the explanted liver confirmed subtotal necrosis of the liver with extensive cholestasis and predominantly lymphocytic hepatitis. Subsequent maternal sonographic controls and laboratory testing showed normal liver function and perfusion. Subsequent biopsies of the transplanted liver due to elevated liver enzymes at day six after transplantation, could exclude a graft rejection. Eleven days after liver transplantation fetal sonography revealed hepatomegaly and intracranial ventriculomegaly (Figure 1(a)). Additionally, small hematomas in the plexus choroideus on both sides and a subdural bleeding in the posterior cranial fossa were detected (Figure 1(b)). Therefore, an additional MRI of the fetus was arranged five days later and confirmed a mild ventriculomegaly on both sides. However, the hepatomegaly could not be confirmed. Repeated sonographic and MRI examinations showed total restitution of the former findings during the course of pregnancy (Figure 1(c)). Because the patient suffered from preterm uterine contractions, tocolytic agents in form of nifedipine 60 mg orally twice a day in an off-label-use were applied and had to be changed to intravenous admission of hexoprenaline at 28 4/7 GW because of ongoing contractions. The patient developed gestational diabetes, which could be well controlled by diet. With 34 GW a CMV reactivation was detected and treated with CMV immunoglobulins intravenously. An elective cesarean delivery was performed at 36 0/7 GW due to increasing discomfort and strong demand of the patient. A healthy male newborn of 2700 g (42. percentile) was delivered with an APGAR score of 6-4-7 and an umbilical artery pH of 7.39. The child was transferred to our neonatal intensive care unit for better surveillance. A fetal hepatitis B and CMV infection could be excluded by serological testing of the fetal blood and urine. The mother's and child's course were uneventful and they were dismissed eight and 20 days after the cesarean section, respectively.

## 3. Discussion

Acute liver failure during pregnancy is a rare but potentially life-threatening disease. Causes of acute liver failure can be pre-existing, pregnancy related, or can occur during pregnancy without being directly related to it (Table 2). Depending on the age of gestation and the viability of the fetus, termination of pregnancy before or during transplantation or maintaining pregnancy has to be discussed with the patient. A multidisciplinary approach is mandatory and the treatment of patients with acute liver failure during pregnancy should take place in a highly qualified and specialized center.

Besides our case, 18 other cases of liver transplantation during pregnancy were described (Table 3). The reasons for transplantation were acute liver failure due to different etiologies (Table 3). All liver transplantation procedures were performed with piggy back technique. According to current guidelines, immunosuppressive treatment mostly consists of a combination of a calcineurin inhibitor (e.g., tacrolimus) and corticosteroids [19, 21, 22], as it was in our patient. She tolerated the medication without any problems.

Described side effects during pregnancy after liver transplantation, when transplantation was performed before the begin of a pregnancy, are hypertension (26–46%), preeclampsia (9–26%), cholestasis (3–27%), graft rejection (7–12%), diabetes (5–13%), osteoporosis, neurotoxicity, impaired maternal renal function, maternal infections (9–27%), cesarean delivery (23–47%), preterm delivery (31–39%), fetal growth restriction (17–34%), indistinct fetal malformations (0–3%), and complications of the newborn (17–33%) with a perinatal mortality of 0–4% [19–24].

Side effects after transplantation during the ongoing pregnancy in the 18 cases differed little, with cholestasis (22%), graft rejections (25%), impaired maternal renal function (6%), maternal infections (13%), cesarean delivery (25%), preterm delivery (44%), fetal growth restriction (22%), complications of the newborn (19%), and perinatal mortality (50%) [1–18]. Concerning our case, maternal complications in form of a steroid induced gestational diabetes, a reactivation of CMV-infection due to the immunosuppressive medication and preterm contractions, well controlled by tocolysis, took place. No serious complications were found. Concerning the fetus, intermittent intracranial ventriculomegaly and intracranial bleeding with a total restitution in the further course of pregnancy were documented. The reason for these intermittent changes was unclear. By treating the mother with antiviral drugs and immunoglobulins against hepatitis B and CMV, an infection of the newborn could be successfully prevented. In the end, a normally sized and developed newborn was delivered almost at term. Its development a year after the birth its absolutely normal and uncomplicated, but the neurological long-term outcome is still unknown. In comparison, fetal outcome in the other 18 cases described in the literature showed a wide range from abortion to delivery of a normal fetus (Table 3).

We conclude that super-urgent liver transplantation due to an acute hepatitis B infection during pregnancy can be a life-saving procedure for the mother and fetus. An early initiated maternal therapy with antiviral drugs and immunoglobulins seems to be safe and able to prevent fetal infection. Although perinatal mortality is high, on one hand, because of intrauterine fetal death in the course and on the other hand, because of induced abortion for fear of fetal complications, maintaining the pregnancy should always be discussed with the mother. As especially our case and few others show, maintaining the pregnancy is an option in case of acute liver failure with the necessity of transplantation and positive outcome is possible. Nevertheless, fetal outcomes after transplantation during pregnancy are diverse and long-term outcomes are deficiently known. But as our case shows, there are good chances of positive outcome for affected patients and their offspring as long as they are treated in highly specialized centers with intense treatment and observation.

## Figures and Tables

**Table 1:** Maternal laboratory values.

	Day of admission, 22 0/7 GW	Day of transplantation, 22 3/7 GW	Two days after transplantation, 22 5/7 GW	Discharge of hospital (110 days after transplantation)
Hb in g/L (117–153)	111	83	64	133
Tc in G/L (143–400)	173	194	137	249
INR (<1.2)	1.6	4.6	1.2	1.1
Factor 5 in % (50–150)	27	15	79	
Bilirubin in mcM/L (<21)	129	184	37	7
Ammoniac in mcM/L (9–30)	17	49	14	
AST in U/L (<35)	4030	1749	347	20
ALT in U/L (10–35)	2775	2272	824	12
GGT in U/L (5–36)	19	17		
HBV-DNA IU/mL (0)	117 × 10^6^	110 × 10^6^	6.2 × 10^6^	<20
HBs-Ag in U/mL (<0.05)	pos		2748.68	0
Anti-HBs U/L (<10)	neg		17	787
HBe-Ag (neg)	pos		neg	
Anti-HBe (neg)	pos		pos	
Anti-HBc-IgM (neg)	pos		pos	

**Table 2:** Causes of acute hepatic failure during pregnancy.

Causes of hepatic failure	Pregnancy related	Nonpregnancy related	Testing
HELLP syndrome	x		Thrombocytes, liver enzymes, hemolysis parameters (LDH, haptoglobin)
Acute fatty liver of pregnancy	x		Long-chain fatty acids (LCHAD), acylcarnitine
Infections		x	Serological blood testing
Intoxications (drugs, medication)		x	Urine/blood testing (qualitative/quantitative drug proof)
Metabolic disorders (e.g., M. Wilson)		x	Special markers (e.g., ceruloplasmin, alpha-1-antitrypsin), biopsy
Tumors		x	Imaging, biopsy, tumor markers (e.g., AFP)
Autoimmune disorders		x	Autoantibodies, biopsy

**Table 3:** Eighteen other cases of liver transplantation during pregnancy in the literature.

Publication	GW at transplantation	Etiology	Maternal outcome	Delivery mode, GW	Fetal outcome
Anders et al. [1]	20	Unknown	Survived	Curettage, 20 GW	Intrauterine fetal death (IUFD)
Catnach et al. [2]	20	Autoimmune	Survived, PPROM	Spontaneous, 28 GW	Survived
Eguchi et al. [3]	15	Unknown	Survived, cytomegaly infection	Curettage, 20 GW	Abortion
Fair et al. [4]	22	Hepatitis B	Survived, Retransplantation	Cesarean, 30 GW	Survived, intrauterine growth restriction (IUGR)
Finlay et al. [5]	17.5	Unknown	Survived	Spontaneous, 28.5 GW	IUFD
Hamilton et al. [6]	21	Hepatitis B	Survived	Spontaneous, 22 GW	IUFD
Jarufe et al. [7]	22	Unknown	Survived, reoperation (biliary stenosis), mild graft reaction, preterm contractions	Spontaneous, 27 GW	Survived, no compromises
Laifer et al. [8]	26	Hepatitis B	Survived, Retransplantation	Cesarean, 28 GW	Neonatal death
Laifer et al. [9]	23	Autoimmune	Survived; infection, renal insufficiency, anemia, thrombozytopenia, hypotension graft reaction	Spontaneous, 23 GW	IUFD
Lo et al. [10]	26	Unknown	Survived	Spontaneous, 26 GW	IUFD 26
Kato et al. [11]	13	Unknown	Survived, reoperation (insufficient biliary anastomosis)	Miscarriage, 13 GW	Miscarriage
Moreno et al. [12]	27	Unknown	Survived	Cesarean, term	Survived
Morris et al. [13]	27	Drug (PTU)	Survived	Spontaneous, 27 GW	Neonatal death
Sequeira et al. [14]	18	Drug (PTU)	Survived	Cesarean, 37 GW	IUGR, microcephaly, oligohydramnion, ventriculomegaly, ischemic enzephalopathy, seizures
Jankovic et al. [15]	13.5	Autoimmune	Survived	Spontaneous, 36 GW	No compromises
Maddukuri et al. [16]	11	Unknown	Survived	Spontaneous, 30 GW	Normally developed at age of 4 years
Simsek et al. [17]	18	Hepatitis A	Survived	Induced abortion 18 GW	Abortion, growth restriction, oligohydramnion
Thornton and Minns [18]	20 5/7	Drug	Survived	Induced abortion	Abortion, hydrops, bilateral ventriculomegaly
